# Supplementary material for: Identification of Oak-Barrel and Stainless Steel Tanks with Oak Chips Aged Wines in Ningxia Based on Three-Dimensional Fluorescence Spectroscopy Combined with Chemometrics
Source: Molecules. 2023 Apr 24;28(9):3688. doi: 10.3390/molecules28093688 (PMC10180402; doi:10.3390/molecules28093688)
Supplement: Supplementary file 1 [file molecules-28-03688-s001.zip › molecules-2259184-supplementary.pdf]

Table S1. Detailed information of wine samples collected from different wineries in Ningxia.

Number	Vintage	Grape Varieties	Aged Type	Producer
1	2022	Cabernet Gernischet	stainless steel tank with oak chips	Ningxia Leirenshou wine industry Company Ltd
2	2022	Merlot	stainless steel tank with oak chips	Ningxia Shaquan Grape Wine Company Ltd
3	2022	Pinot Noir	stainless steel tank with oak chips	Yuma International Grape (Ningxia) Wine Company Ltd
4	2022	Pinot Noir	stainless steel tank with oak chips	Yuma International Grape (Ningxia) Wine Company Ltd
5	2022	Cabernet Sauvignon、Marselan	stainless steel tank with oak chips	Ningxia Hejinzun Winery Company Ltd
6	2022	Cabernet Sauvignon	stainless steel tank with oak chips	Ningxia Weijiani Winery Company Ltd
7	2022	Cabernet Franc	stainless steel tank with oak chips	Ningxia Helan Saibei Leqi Winery Company Ltd
8	2022	Cabernet Sauvignon	stainless steel tank with oak chips	Ningxia Zishang Grape Winery Company Ltd
9	2022	Pinot Noir	stainless steel tank with oak chips	Ningxia Hengsheng Xixiawang wine industry Company Ltd
10	2022	Unknown	stainless steel tank with oak chips	Baole Lijia (Ningxia) Wine Brewing Company Ltd
11	2022	Cabernet Gernischet	stainless steel tank with oak chips	Yuma International Grape (Ningxia) Wine Company Ltd
12	2022	Syrah	stainless steel tank with oak chips	Ningxia Lilan Winery Company Ltd
13	2022	Merlot	stainless steel tank with oak chips	Ningxia Hengsheng Xixiawang wine industry Company Ltd
14	2022	Cabernet Gernischet	oak-barrel	Ningxia Zishang Grape Winery Company Ltd

15	2022	Merlot	stainless steel tank with oak chips	Baole Lijia (Ningxia) Wine Brewing Company Ltd
16	2022	Pinot Noir	stainless steel tank with oak chips	Ningxia Hengsheng Xixiawang wine industry Company Ltd
17	2022	Cabernet Sauvignon	stainless steel tank with oak chips	Ningxia Xige Winery Company Ltd
18	2022	Cabernet Sauvignon	stainless steel tank with oak chips	Ningxia Xige Winery Company Ltd
19	2022	Pinot Noir	stainless steel tank with oak chips	Yuma International Grape (Ningxia) Wine Company Ltd
20	2022	Cabernet Sauvignon	stainless steel tank with oak chips	Ningxia Leirenshou wine industry Company Ltd
21	2022	Merlot	stainless steel tank with oak chips	Ningxia Xixia King Wine Industry Company Ltd
22	2022	Pinot Noir	stainless steel tank with oak chips	Ningxia Xixia King Wine Industry Company Ltd
23	2022	Cabernet Sauvignon	stainless steel tank with oak chips	Ningxia Heilanting Winery Company Ltd
24	2022	Cabernet Sauvignon	stainless steel tank with oak chips	Ningxia Xige Winery Company Ltd
25	2022	Cabernet Sauvignon	oak-barrel	Ningxia Shiyu Winery Company Ltd
26	2022	Cabernet Sauvignon	stainless steel tank with oak chips	Ningxia Heilanting Winery Company Ltd
27	2022	Cabernet Sauvignon	stainless steel tank with oak chips	Ningxia Xige Winery Company Ltd
28	2022	Merlot、Cabernet Sauvignon	stainless steel tank with oak chips	Baole Lijia (Ningxia) Wine Brewing Company Ltd
29	2022	Cabernet Sauvignon	oak-barrel	Ningxia Xige Winery Company Ltd
30	2022	Merlot	oak-barrel	Ningxia Ganchengzi Wine Industry Company Ltd

31	2022	Merlot	stainless steel tank with oak chips	Ningxia Xixia King Wine Industry Company Ltd
32	2022	Cabernet Sauvignon	oak-barrel	Ningxia Fuhai Wine Company Ltd
33	2022	Cabernet Sauvignon	stainless steel tank with oak chips	Ningxia Shiyu Winery Company Ltd
34	2022	80% of Merlot、10% of Syrah、10% of Marselan	stainless steel tank with oak chips	Ningxia Huangkou Winery Company Ltd
35	2022	Cabernet Gernischet	stainless steel tank with oak chips	Ningxia Yuhuang Winery Company Ltd
36	2022	70% of Cabernet Sauvignon、20% of Cabernet Gernischet、5% of Merlot	oak-barrel	Ningxia Luoshan Winery Company Ltd
37	2022	Cabernet Sauvignon	stainless steel tank with oak chips	Ningxia shaquan grapes brewing Company Ltd
38	2022	Pinot Noir	stainless steel tank with oak chips	Ningxia Xixia King Wine Industry Company Ltd
39	2022	Pinot Noir	stainless steel tank with oak chips	Ningxia Zishang Grape Winery Company Ltd
40	2022	70% of Cabernet Sauvignon、20% of Cabernet Gernischet、5% of Merlot	oak-barrel	Ningxia Luoshan Winery Company Ltd
41	2022	80% of Merlot、20% of Syrah	oak-barrel	Ningxia Leirenshou wine industry Company Ltd
42	2022	Cabernet Sauvignon	stainless steel tank with oak chips	Ningxia Huida Sunshine Ecological Winery Company Ltd
43	2022	Cabernet Sauvignon	stainless steel tank with oak chips	Ningxia Danlu Winery Company Ltd
44	2022	Cabernet Sauvignon	stainless steel tank with oak chips	Ningxia Danlu Winery Company Ltd
45	2022	Cabernet Sauvignon	stainless steel tank with oak chips	Ningxia Yuhuang Winery Company Ltd
46	2022	Pinot Noir	stainless steel tank with	Ningxia Shengxin Winery Company Ltd

			oak chips	
47	2022	Cabernet Sauvignon、Merlot、Cabernet Franc	stainless steel tank with oak chips	Ningxia Xixia King Wine Industry Company Ltd
48	2022	Cabernet Gernischet	stainless steel tank with oak chips	Guangxia (Yinchuan) Industrial Company Ltd
49	2022	Cabernet Sauvignon	oak-barrel	Ningxia Xige Winery Company Ltd
50	2022	Cabernet Gernischet	stainless steel tank with oak chips	Ningxia Xige Winery Company Ltd
51	2022	Cabernet Sauvignon	stainless steel tank with oak chips	Baole Lijia (Ningxia) Wine Brewing Company Ltd
52	2022	Unknown	stainless steel tank with oak chips	Baole Lijia (Ningxia) Wine Brewing Company Ltd
53	2022	Cabernet Sauvignon	stainless steel tank with oak chips	Ningxia Xige Winery Company Ltd
54	2022	70% of grape、30% of Wolfberry	stainless steel tank with oak chips	Ningxia hongzhongning wolfberry Company Ltd
55	2022	Pinot Noir	stainless steel tank with oak chips	Ningxia hongzhongning wolfberry Company Ltd
56	2022	Unknown	oak-barrel	Ningxia langfei wine Company Ltd
57	2022	Unknown	stainless steel tank with oak chips	COFCO Great Wall Wine (Ningxia) Company Ltd
58	2022	Unknown	stainless steel tank with oak chips	Baole Lijia (Ningxia) Wine Brewing Company Ltd
59	2022	Cabernet Sauvignon、Merlot	stainless steel tank with oak chips	Baole Lijia (Ningxia) Wine Brewing Company Ltd
60	2022	Cabernet Sauvignon	stainless steel tank with oak chips	Ningxia Red Wolfberry Industry Company Ltd
61	2022	Cabernet Sauvignon	oak-barrel	Ningxia Shaquan Grape Winery Company Ltd
62	2022	Cabernet Sauvignon	stainless steel tank with oak chips	Ningxia Ganchengzi Wine Industry Company Ltd

63	2022	Cabernet Sauvignon、 Cabernet Gernischet	oak-barrel	Ningxia Xige Winery Company Ltd
64	2022	Cabernet Gernischet	oak-barrel	Ningxia Zishang Grape Winery Company Ltd
65	2022	Cabernet Sauvignon	oak-barrel	Ningxia Shiyu Winery Company Ltd
66	2022	Cabernet Sauvignon	oak-barrel	Ningxia Xige Winery Company Ltd
67	2022	Merlot	oak-barrel	Ningxia Ganchengzi Wine Industry Company Ltd
68	2022	Cabernet Sauvignon	oak-barrel	Ningxia Fuhai Wine Company Ltd
69	2022	70% of Cabernet Sauvignon、20% of Cabernet Gernischet、5% of Merlot	oak-barrel	Ningxia Luoshan Winery Company Ltd
70	2022	70% of Cabernet Sauvignon、20% of Cabernet Gernischet、5% of Merlot	oak-barrel	Ningxia Luoshan Winery Company Ltd
71	2022	80% of Merlot、20% of Syrah	oak-barrel	Ningxia Leirenshou wine industry Company Ltd
72	2022	Cabernet Sauvignon	oak-barrel	Ningxia Xige Winery Company Ltd
73	2022	Unknown	oak-barrel	Ningxia langfei wine Company Ltd
74	2022	Cabernet Sauvignon	oak-barrel	Ningxia Shaquan Grape Winery Company Ltd
75	2022	Cabernet Sauvignon、 Cabernet Gernischet	oak-barrel	Ningxia Xige Winery Company Ltd
76	2022	Cabernet Gernischet	oak-barrel	Ningxia Zishang Grape Winery Company Ltd
77	2022	Cabernet Sauvignon	oak-barrel	Ningxia Shiyu Winery Company Ltd
78	2022	Cabernet Sauvignon	oak-barrel	Ningxia Xige Winery Company Ltd
79	2022	Merlot	oak-barrel	Ningxia Ganchengzi Wine Industry Company Ltd
80	2022	Cabernet Sauvignon	oak-barrel	Ningxia Fuhai Wine Company Ltd
81	2022	70% of Cabernet Sauvignon、20% of Cabernet Gernischet、5% of Merlot	oak-barrel	Ningxia Luoshan Winery Company Ltd
82	2022	70% of Cabernet Sauvignon、20% of Cabernet Gernischet、5% of Merlot	oak-barrel	Ningxia Luoshan Winery Company Ltd
83	2022	80% of Merlot、20% of Syrah	oak-barrel	Ningxia Leirenshou wine industry Company Ltd

84	2022	Cabernet Sauvignon	oak-barrel	Ningxia Xige Winery Company Ltd
85	2022	Unknown	oak-barrel	Ningxia langfei wine Company Ltd
86	2022	Cabernet Sauvignon	oak-barrel	Ningxia Shaquan Grape Winery Company Ltd
87	2022	Cabernet Sauvignon、 Cabernet Gernischet	oak-barrel	Ningxia Xige Winery Company Ltd
